# Toll-like receptor 2 signaling pathway activation contributes to a highly efficient inflammatory response in Japanese encephalitis virus-infected mouse microglial cells by proteomics

**DOI:** 10.3389/fmicb.2022.989183

**Published:** 2022-09-12

**Authors:** Guanyu Zhao, Yan Gao, Jiaqi Zhang, He Zhang, Changzhan Xie, Fulong Nan, Sheng Feng, Zhuo Ha, Chenghui Li, Xiangyu Zhu, Zhuoxin Li, Ping Zhang, Ying Zhang, Huijun Lu, Ningyi Jin

**Affiliations:** ^1^College of Veterinary Medicine, College of Animal Science, Jilin University, Changchun, China; ^2^Changchun Veterinary Research Institute, Chinese Academy of Agricultural Sciences, Changchun, China; ^3^Research Unit of Key Technologies for Prevention and Control of Virus Zoonoses, Chinese Academy of Medical Sciences, Changchun, China

**Keywords:** Japanese encephalitis virus (JEV), toll-like receptor 2 (TLR2), proteomics, inflammatory response, microglial

## Abstract

Thousands of people die each year from Japanese encephalitis (JE) caused by the Japanese encephalitis virus (JEV), probably due to exacerbation of the inflammatory response that impairs the course of the disease. Microglia are mononuclear phagocytic cells located within the parenchyma of the central nervous system; these play a key role in the innate immune response against JEV infections. However, the involvement of toll-like receptor 2 (TLR2) in the inflammatory response during the early stages of JEV infection in BV2 cells remains. Here, we evaluated protein profiles and determined the role of TLR2 in the inflammatory response of JEV-infected BV2 cells. High-depth tandem mass tags labeling for quantitative proteomics was used to assess JEV infected-BV2 cells and compare immune response profiles at 6, 12, and 24 h post-infection (hpi). In total, 212 upregulated proteins were detected at 6 hpi, 754 at 12 h, and 191 at 24 h. According to GO and KEGG enrichment analysis, the upregulated proteins showed enrichment for proteins related to the immune response. Parallel reaction monitoring tests, western blotting, and qPCR results showed that the adaptor protein MyD88 was not activated. The expression levels of key proteins downstream of MyD88, such as IRAK1, IRAK4, and TRAF6 did not increase; however, the expression levels of PI3K-AKT did increase. By inhibiting key proteins (TLR2, PI3K, and AKT) we confirmed that JEV activated TLR2, thus resulting in a robust inflammatory response. Consequently, the TLR2-PI3K-AKT signaling axis was proven to play a critical in the early stages of the JEV infection-induced inflammatory response in microglia.

## Introduction

Japanese encephalitis virus (JEV) belongs to the family Flaviviridae (Seo et al., 2013). Although JEV is endemic to many parts of the world, its prevalence is high in developing countries in the Asian subcontinent (Solomon, 2006).

Japanese encephalitis virus crosses the blood-brain barrier and causes damage to the central nervous system (CNS; Wang et al., 2018). Microglia are the resident macrophages in the parenchyma of the CNS and serve as the first line of defense against invading pathogens, playing an important role in the inflammatory response. When pathogens invade, microglia are rapidly activated, and inflammatory cytokines are released to mediate the inflammatory response. The massive activation of microglia is one of the hallmark features of JEV infection in the brain. During JEV infection of the microglia, the release of several inflammatory factors, such as iNOS, TNF-α, IL-1β, and CCL3, triggers an inflammatory response that exacerbates neuroinflammation and neuronal cell death (Lannes et al., 2017). These inflammatory responses are induced by pattern recognition receptors (PRRs) on the surface of the microglia, including Toll-like receptors that recognize pathogen-associated molecular patterns (PAMPs). Toll-like receptors detect and destroy pathogens that invade the CNS parenchyma (Mariani and Kielian, 2009) and trigger the production of inflammatory mediators (Poeck and Ruland, 2012).

Previous reports suggested that JEV infection induces an inflammatory response *via* TLR3 (Jiang et al., 2014). However, whether JEV infection causes an inflammatory response only through the TLR3 pathways is a matter of concern. Previous research has shown that TLR2 mediates a parasite-induced inflammatory response (Wei et al., 2021), although the mechanism underlying the TLR2-mediated inflammatory response in JEV infection has yet to be elucidated. The activation of TLR2 and its downstream pathways following JEV infection have still to be investigated. Neurotropic flaviviruses, including Japanese encephalitis, are able to participate in the body’s immune response through the TLR signaling pathway. The interaction of TLR2 with the PI3K/AKT pathway has been demonstrated in many fields, including the regulation of inflammatory response and autophagy (Jiang et al., 2017), protection against acute kidney injury (Shen et al., 2019), protein synthesis (Jing et al., 2016), and the activation of antigen-presenting cells and T cells (Cho et al., 2018). The innate immune response also plays a crucial role in the initial control of JEV infection and causes an inflammatory process (Paun and Pitha, 2006). However, current studies do not describe the innate immune pathways that cause inflammatory responses in microglia infected by JEV.

In this study, we applied proteomics technology to comprehensively identify proteins that were activated in the microglia after JEV infection. We investigated the expression of TLR2, which undergoes significant changes in expression during JEV infection, and validated its downstream PI3K-AKT pathway to fully describe its role in activating the inflammatory response. Our study provides further understanding of JEV-induced microglial cell inflammation and provides a basis for preventing inflammation-induced neuronal damage in the future by providing options to reduce intracerebral damage after JEV infection.

## Materials and methods

### Cell culture

BV2 cells (CCTCC, China) were cultured in DMEM media (Gibco, Grand Island, NY, United States) containing 5% FBS (Gibco) in an incubator at 37°C.

### Japanese encephalitis virus titration and infection

Japanese encephalitis virus titration was performed using BHK cells as described elsewhere (Xiao et al., 2018). BV2 cells were uniformly seeded at a density of 6 × 10^5^ cells/well. BV2 were then infected with JEV at a multiplicity of infection (MOI) of 1 for 2 h; then DMEM culture media was added. Cells were collected at 6, 12, and 24 h and kept at −80°C until use.

### Protein extraction and LC-MS/MS

Protein extraction, LC-MS/MS, LC-MS/MS Analysis, and PRM tests were performed using previously described protocols (Li et al., 2021; Yan et al., 2021). Further details related to these protocols are shown in Supplementary Table 1.

### RNA extraction and quantitative real-time PCR

Total RNA was extracted from microglia using TRIzol reagent in the BV2 mock, BV2_6h, BV2_12h, and BV2_24h groups, in accordance with the manufacturer’s recommendations (Sangon Biotech, Shanghai, China). Reverse transcription was then performed with a Reverse Transcription Kit (TaKaRa Biotechnology, Dalian, China). An ABI 7500 Real Time PCR System was then used to perform quantitative real-time PCR (RT-PCR) using cDNA as the template for SYBR green-based qPCRs. The threshold cycle (2^–ΔΔCt^) approach was used to calculate fold changes in transcript levels (Pfaffl, 2001), and GAPDH expression was used to standardize the values. The qPCR primers used in this study are listed in Supplementary Table 1.

### Western blotting

Cells were rinsed in PBS and scraped in 400 ml of cell lysis buffer containing protease and phosphatase inhibitor cocktails (Roche, Mannheim, Germany). Cell lysates were then centrifuged at 12,000 rpm for 15 min at 4°C. The protein concentration in each lysate was then determined using the BCA assay. SDS-PAGE gels were loaded with the same amount of protein, and the NC membrane was blocked for 30 min with skimmed milk. Several primary antibodies were used for western blotting (as 1:1000 dilutions): NF-κB p65, ab16502; IkBα, ab76429; TLR2, ab108998; MyD88, ab219413; IRAK1, ab238; TRAF6, ab33915; TIRAP, ab17218; TAK1, ab109526; p-NF-κB p65, ab76302; IRAK4, 4363; p-PI3K, ab182651 (Abcam, Cambridge, MA, United States). We also used β-actin, 4970, GAPDH, 5174; AKT, 9272; and p-AKT, 4060S (Cell Signaling Technology, United States). HRP-labeled goat anti-rabbit secondary antibodies were used at a dilution of 1:2000 (A0208; Beyotime Biotechnology, Shanghai, China) and immunoreactive bands were detected using an Amersham Imager 600 (GE Healthcare Bio-Sciences AB, Uppsala, Sweden).

### Protein inhibiting

Toll-like receptor 2 was inhibited by C29 (MedChemExpress, Monmouth Junction, NJ, United States) at 150 μM for 60 min, while PI3K was inhibited by 3-Methyladenine (MCE) at 10 mM for 1 h. AKT was inhibited by MK 2206 (MCE) at 10 μM for 2 h; subsequently, JEV incubation took place for 12 h with an MOI of 1.

### Statistical analysis

The student’s *t*-test was used to analyze differences between groups after JEV infection (GraphPad PRISM, San Diego, CA, United States).

## Results

### The proteome was expressed differentially in BV2 cells following Japanese encephalitis virus infection

**FIGURE 1:**
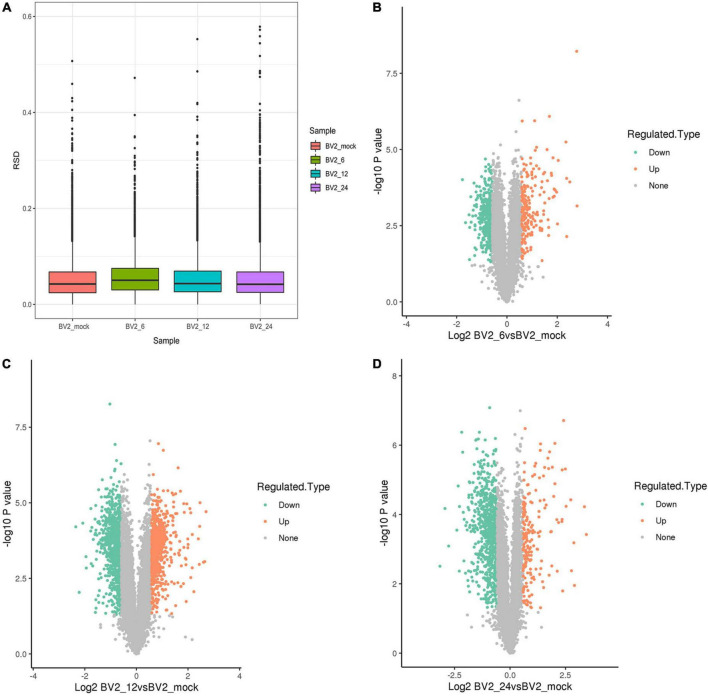
**(A)** Boxplots illustrating relative standard deviation (RSD) of protein quantification results across replicates, with lower overall RSD values indicating higher quantitative reproducibility. **(B)** 212 upregulated DEPs at 6 hpi. **(C)** 754 upregulated DEPs at 12 hpi. **(D)** 191 upregulated DEPs at 24 hpi.

We performed a proteome-wide study of DEPs by MS in BV2 cells infected with JEV at 6 h post infection (hpi), 12 hpi, 24 hpi, and in a mock group. Boxplots were used to depict the relative standard deviation (RSD) of protein quantification data across replicates, with lower overall RSD values indicating greater quantitative reproducibility (Figure 1A).

Following quality checks and data filtering, 7,493 proteins were identified (Supplementary Table 2). During JEV infection, we identified 212 upregulated DEPs and 430 downregulated DEPs at 6 hpi, 754 upregulated DEPs and 927 downregulated DEPs at 12 hpi, and 191 upregulated DEPs and 778 downregulated DEPs at 24 hpi (Supplementary Table 3 and Figures 1B–D).

### Functional characterization of differentially expressed proteins

**FIGURE 2:**
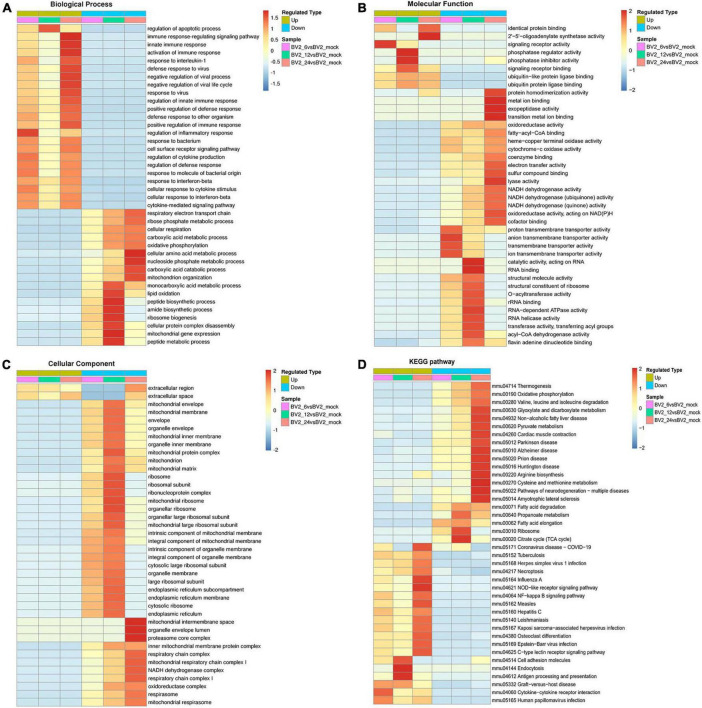
Functional enrichment of DEPs in BV2 cells after JEV infection. **(A–C)** Heat map of cluster analysis based on GO enrichment. **(A)** Biological process showing activation of multiple immune responses, indicating activation of cellular immune responses after JEV infection. **(B)** Molecular function showing activation associated with protein binding, suggesting that the cascade effect of the immune response is also activated. **(C)** The cellular component was enriched for fewer upregulated proteins and a larger number of proteins with decreased expression, thus indicating that intracellular component expression was suppressed by JEV infection. **(D)** Heat map of clustering analysis for KEGG enrichment. Infectious disease pathways were activated, while the expression of proteins related to autoimmune disease-related pathways was decreased. Cell adhesion molecules, along with antigen processing and presentation pathways, were highly enriched at 12 h. All enriched proteins were significantly different, with a red to blue gradient indicating higher to lower protein enrichment.

In the biological process category (Figure 2A), the upregulated DEPs were found to be primarily associated with the regulation of inflammatory responses, cellular response to cytokine stimulus, regulation of apoptotic process, innate immune response, and signaling receptor activity (Figure 2B). In the cellular component category, the upregulated DEPs were primarily associated with the extracellular region and the extracellular space (Figure 2C). DEPs in the BV2_12h/mock group were enriched in cell adhesion molecules, antigen processing, and antigen presentation (Figure 2D) and in the Toll-like receptor and PI3K-AKT signaling pathways. The candidate peptides of the 15 target proteins in the BV2_12h/mock group were analyzed using LC-PRM. TLR2 expression increased at 12 hpi (Table 1), whereas MyD88, the TLR receptors general downstream protein, was not upregulated at this time.

**TABLE 1:** PRM validation of the proteomics.

Protein accession	Gene name	Mean of BV2_mock PRM/TMT ratio	Mean of BV2_6h PRM/TMT ratio	Mean of BV2_12h PRM/TMT ratio	Mean of BV2_24h PRM/TMT ratio
Q05769	Ptgs2	0.24	1.53**	1.16**	1.07**
P27512	Cd40	0.00	0.93**	1.52**	0.55**
P10810	Cd14	0.35	1.26**	1.86**	0.53**
P13597	Icam1	0.20	1.29**	2.08**	0.43**
Q9WTK5	NF-κB2	0.47	1.05**	1.14**	1.34**
Q9Z0H7	Bcl10	0.40	1.10**	1.50**	1.00**
P42225	Stat1	0.24	0.79**	1.26**	1.71**
Q9QUN7	Tlr2	0.46	1.22**	0.94**	1.37**
O35613	Daxx	0.33	1.04**	1.47**	1.15**
Q64339	Isg15	0.04	1.09**	2.26**	0.62**
Q9EQ32	Pik3ap1	0.51	1.14**	1.15**	1.20**
O88522	Ikbkg	0.73	0.88-	1.51**	0.89-
P25799	Nfkb1	0.43	1.19**	1.08**	1.30**
P02468	Lamc1	0.69	1.22**	1.50**	0.59-
P22366	MyD88	0.82	0.93-	0.73-	1.53**

-, not significantly regulated, **p < 0.01.

**FIGURE 3:**
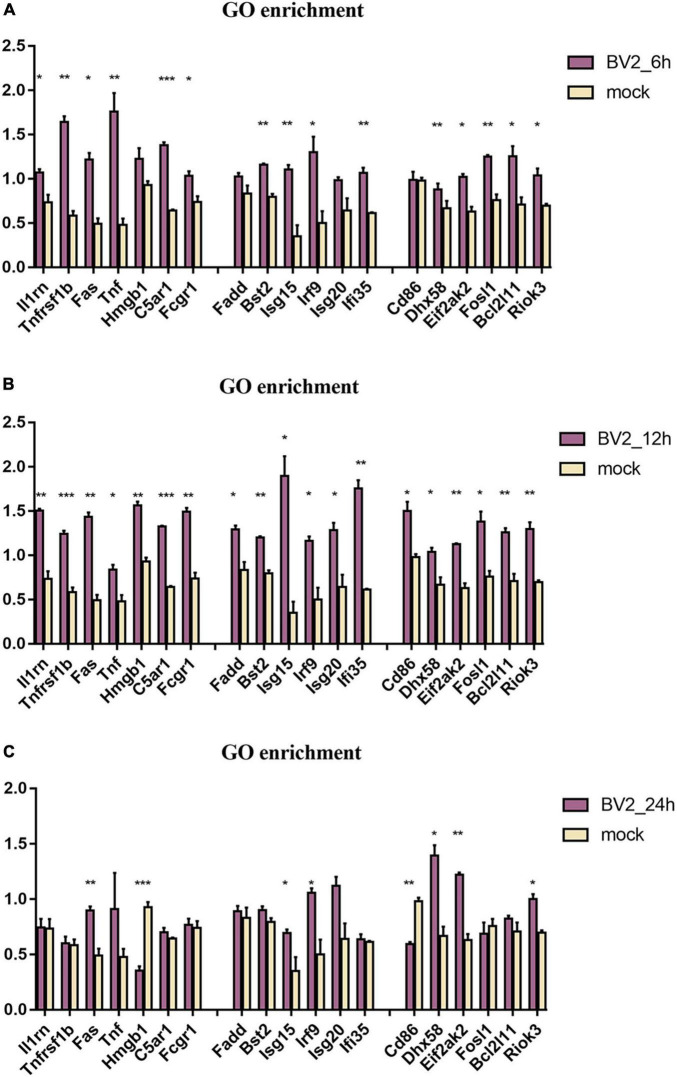
Regulation of proteins associated with immune in BV2 cells after infection with JEV at **(A)** 6 hpi, **(B)** 12 hpi, and **(C)** 24 hpi. Significant changes were found in the expression of inflammation-related proteins Fas, TNF, Fosl1, Hmgb1, Riok3, as well as apoptosis-related proteins Fadd, Fas, TNF, and interferon-related proteins Irf9, Isg20, Ifi35. These suggested that JEV infection induces broad immune responses, with the inflammatory response being one of the key immune responses. All the samples were tested through three parallel groups. *P < 0.05, **P < 0.01, and ***P < 0.001.

By using GO enrichment analysis, we discovered numerous inflammatory responses, innate immune responses, and antiviral response-related proteins at 6, 12, and 24 hpi (Figure 3). We observed a significant upregulation of proteins at 12 hpi than at any other time point (Figure 3B).

### Japanese encephalitis virus induced a strong inflammatory response and associated proteins were identified in BV2 cells

**FIGURE 4:**
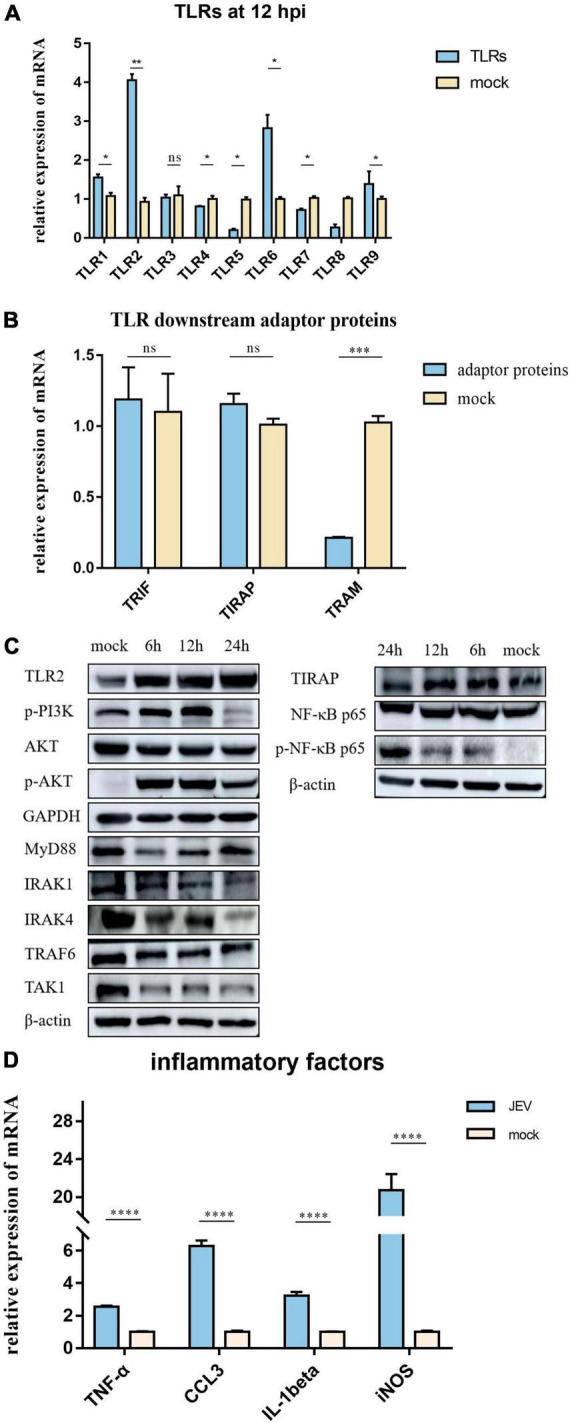
According to proteomic data, the most immune-related proteins were activated 12 h after infection. **(A)** Detection of TLR activation by relative mRNA level. TLR2 shows highlight expression in all TLRs; TLR2 was selected for subsequent investigation. **(B)** To investigate the pathway by which TLR2 exerts functionality, qPCR was performed on critical proteins downstream of TLR2; TRIF, TIRAP, and TRAM were not upregulated. **(C)** Two pathways downstream of TLR2 were verified by western blotting. The expression levels of PI3K-AKT and NF-κB pathway proteins were significantly upregulated; however, the MyD88 pathway, which is usually considered a TLR2 junction protein, was not activated. **(D)** JEV infection promoted the upregulation of inflammatory factors (TNF-α, CCL3, IL-1beta, and iNOS), thus indicating an inflammatory response. All samples were tested in three parallel groups. *P < 0.05, **P < 0.01, ***P < 0.001, and ****P < 0.0001.

The expression levels of TLR1, TLR2, TLR3, TLR4, TLR5, TLR6, TLR7, TLR8, and TLR9 were measured by qPCR (Figure 4A). In addition, TLR2 expression was elevated at the protein level, as verified by PRM (Table 1) and western blotting (Figure 4C). TLR2 key adapter proteins and downstream signaling pathway proteins were also detected (Table 1 and Figures 4B,C); the levels of IRAK4, IRAK1, TRAF6, and TAK1 remained unchanged and the MyD88 signaling pathway was not activated. The expression levels of key proteins (TRIF, TIRAP, and TRAM) were not found to be elevated. However, the expression levels of PI3K, AKT, and NF-κB were increased. The expression of inflammatory factors (Figure 4D), including TNF-α, CCL3, IL-1beta, and iNOS, were also increased 12 h after JEV infection.

### Toll-like receptor 2 increased the levels of inflammatory factors by regulating the PI3K-AKT pathway

**FIGURE 5:**
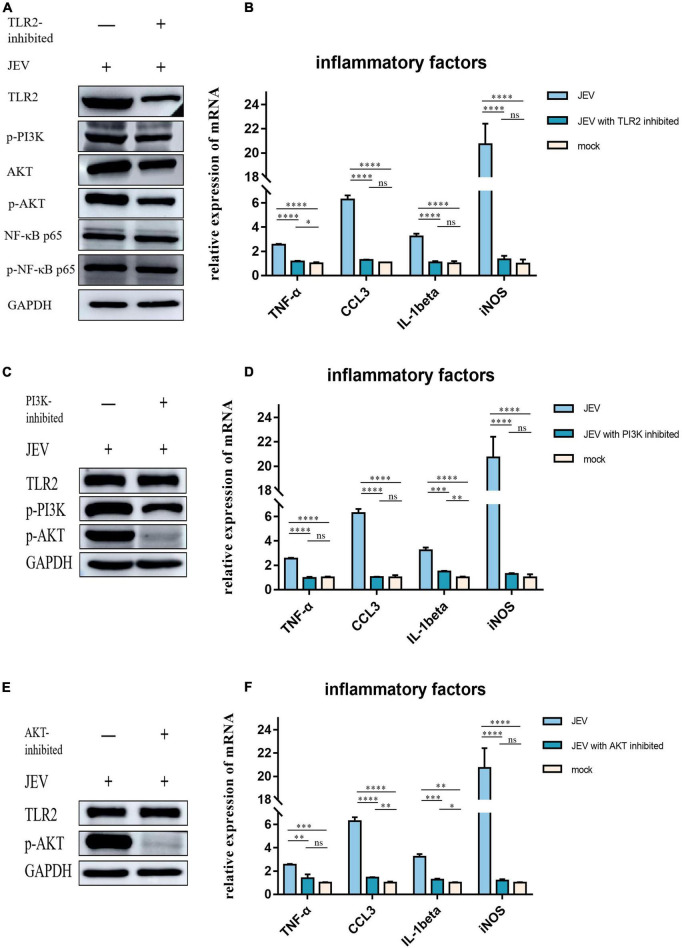
Regulation of inflammatory factor expression by TLR2 through PI3K and AKT was determined by inhibition of key proteins in the pathway. **(A)** We subjected TLR2 to inhibition and examined the downstream key proteins (p-PI3K, p-AKT, and NF-κB). p-PI3K and p-AKT expression decreased with TLR2 expression, whereas NF-κB and p-NF-κB did not change with TLR2 expression; these results suggested that PI3K and AKT are downstream of TLR2 and are regulated by regulation of TLR2, rather than the NF-κB pathway. **(B)** The expression of key inflammatory factors (TNF-α, CCL3, IL-1beta, and iNOS) were significantly decreased after TLR2 inhibition, thus indicating that TLR2 is a key protein in the regulation of inflammation. **(C)** PI3K, a key protein downstream of TLR2, was inhibited and the expression of TLR2 remained unchanged, while the expression of AKT was decreased, thus indicating that PI3K acts downstream of TLR2. **(D)** The expression of key inflammatory factors all decreased after PI3K inhibition. **(E)** AKT was inhibited but this did not induce changes in TLR2 expression, thus indicating that AKT is a downstream protein of TLR2. **(F)** The expression levels of TNF-α, CCL3, IL-1beta, and iNOS were all decreased after AKT inhibition. These results suggest that the expression of inflammatory factors is regulated by the TLR2-PI3K-AKT signaling axis. ns: not signifificantly regulated, *P < 0.05, **P < 0.01, ***P < 0.001, and ****P < 0.0001.

To investigate the role of TLR2 as a key receptor for activating inflammation during JEV infection, we used C29 to inhibit TLR2 expression in BV2 cells (Figure 5A). We also detected the expression levels of candidate proteins downstream of TLR2. The expression levels of p-PI3K and p-AKT decreased while the expression levels of NF-κB and p-NF-κB remained unchanged. Next, we investigated the expression levels of inflammatory factors in BV2 cells at JEV 12 hpi. The expression of inflammatory factors in JEV-infected BV2 cells decreased after TLR2 inhibition (Figure 5B).

PI3K inhibition was followed by a simultaneous reduction in p-AKT expression (Figure 5C) while TLR2 expression remained unchanged. We also investigated the expression levels of TNF-α, CCL3, IL-1beta, and iNOS, which all decreased with PI3K inhibition (Figure 5D).

To determine the role of AKT in the regulation of inflammatory factors in the TLR2-PI3K-AKT signaling axis, we inhibited AKT and examined the expression levels of TLR2 (Figure 5E). The expression of inflammatory factors was also observed to decrease when AKT was inhibited (Figure 5F).

**FIGURE 6:**
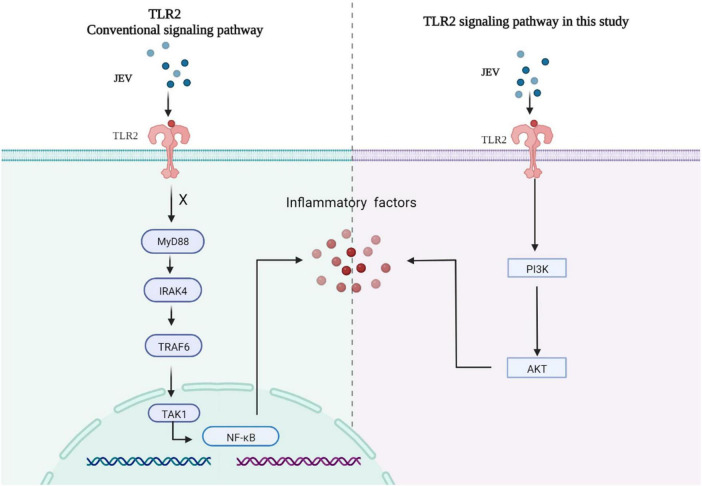
JEV infection increased the expression of key factors of the inflammatory response via the TLR2-PI3K-AKT signaling axis. The MyD88 signaling pathway downstream of TLR2 was not activated, and the NF-κB signaling pathway was not regulated by TLR2.

Collectively, high-depth TMT proteomics was used to detect the innate immune activation of JEV infection over time *in vitro*. We discovered that JEV activates an inflammatory response via the TLR2-PI3K-AKT axis (Figure 6).

## Discussion

Reactive gliosis, an unregulated inflammatory response, along with neuronal cell death, are considered the key hallmarks of JEV infection in the CNS. The significant activation of glial (microglia and astrocytes) and neuronal cells after JEV enters the brain is a characteristic feature of JE. Several pathways are activated in the microglia, astrocytes, and neurons to initiate the neuroinflammatory response. Microglia are the resident macrophage population in the CNS parenchyma and serve as the initial line of defense against invading pathogens. Microglia have a diverse set of pattern recognition receptors, including members of the Toll-like receptor and phagocytic receptor families; these work together to detect and remove microorganisms that have infiltrated the CNS parenchyma (Mariani and Kielian, 2009). TLR2 is an innate immune sensor on the cell surface that may identify ligands from viruses, fungi, bacteria, and parasites (Wei et al., 2021). TLR2 has been found to sense various viral proteins after infection, including Epstein-Barr virus dUTPase, cytomegalovirus glycoprotein B, and hepatitis B virus capsid (Wei et al., 2021). JEV induces neuroinflammation, which has been identified as a significant element in JEV pathogenesis in humans, including immune cell infiltration and neuronal degeneration (Misra and Kalita, 2010).

Similar proteomic results were detected at 6 and 12 hpi, although we did detect fewer differential proteins at 6 hpi. Therefore, the results at 12 hpi may more closely represent the landscape of JEV infection with BV2. MyD88 was significantly activated at 24 hpi, thus suggesting a different innate immune pathway than that at 12 hpi; this will need to be investigated further in future experiments. GO analysis yields information relating to the biological processes, molecular functions, and cellular components that are enriched by DEPs (Li et al., 2017). Because the host inflammatory response is one of the most critical features of JEV-microglia interactions, we focused on microglia immune response-related proteins. JEV infection activates the innate immune response (Zhou et al., 2018) and the antiviral response (Ye et al., 2017), thus resulting in an inflammatory response. Our proteomic results demonstrated enrichment for a wide range of immune-related proteins. The inflammatory response-associated proteins Fas and TNF are apoptotic signaling molecules and Hmgb1 is one of the most important inflammatory mediators of the lethal effects of endotoxin. The innate immune response-associated protein Fadd promotes apoptosis in cells where Fas proteins are located, whereas Irf9, Isg20, and Ifi35 are interferon-associated proteins. The virus-associated protein Fosl1 has a role in regulating apoptosis and inflammatory responses, whereas Riok3 has a regulatory role in macrophages for type I interferons and inflammatory factors. At 12 h after infection, JEV activated antiviral immune responses, inflammatory responses, and innate immune responses, and many related proteins were elevated, thus implying that this is a critical time point for the immune reaction.

In the present study, we describe the global proteome characterization of BV2 responses to JEV infection at different time points by applying TMT quantitative proteomics. Furthermore, we discovered that at 12 hpi, there was a significant increase in the expression levels of PRRs and critical inflammatory pathway-related proteins, by applying quantitative proteomics, qPCR, and western blotting. We identified the activation of TLR2 as a key factor in the production of strong inflammatory factors in BV2 cells. This is consistent with the results of JEV infection in N9 cells (Sharma et al., 2021). In addition, TLR2 was shown to be activated in specimens taken from patients with Rasmussen encephalitis; TLR2 was expressed at low levels in the surgical control cortex group, thus providing direct evidence for TLR2-mediated chronic inflammation in the brain (Luan et al., 2016). Similarly, TLR2-dependent signaling promotes the production of pro-inflammatory cytokines during coronavirus infection. TLR2 detects the SARS-CoV-2 envelope protein and promotes the production of proinflammatory cytokines during coronavirus infection in bone marrow-derived macrophages (Zheng et al., 2021). It has also been found that the stimulation of SARS-CoV-2 S protein can also activate TLR2 and cause a strong inflammatory response in HEK293T cells and A549 cells (Khan et al., 2021). In general, the activation of both MyD88 and NF-κB is essential for TLR2-mediated inflammatory responses following viral infection. TLR2 requires the adaptor protein MyD88 to activate IRAK and TRAF6, sometimes assisted by TIRAP. TRAF6 triggers the activation of TAK, which inhibits phosphoprotein IκB and then activates transcription factor NF-κB. For instance, the inhibition of TLR2 downregulates the MyD88 and NF-κB pathways and reduces inflammatory factor expression in HSV1 encephalitis-infected BV2 cells (Guo et al., 2015). However, our results show that these proteins are not activated after JEV infection. By regulating the key proteins downstream of TLR2, we confirmed that the TLR2-PI3K-AKT signaling axis is the key to regulate the inflammatory response in JEV infection. In confirming that TLR2 was the most obviously activated protein in TLRs, we found that TLR6 was activated, although weaker than TLR2. TLR6 may form heterodimers with TLR2, which can recognize viral proteins and enhance infection, leading to increased TLR6 expression (Henrick et al., 2015). However, TLR2/6 heterodimers do not co-precipitate in human cytomegalovirus (HCMV) infection (Boehme et al., 2006). Also, TLR6 is found to act on inflammation and autophagy in macrophages, but via the MyD88 signaling pathway and NF-κB signaling pathway (Roy et al., 2014). These results suggest that TLR6 has a complex function and may play an essential role in various diseases and inflammation. However, the elevated expression of TLR6 during JEV infection remains puzzling, and more evidence is needed.

Toll-like receptor 2, a type of TLRs, has the ability to recognize pathogens (de Aquino et al., 2014) and endogenous alarm proteins (Thierry et al., 2014) and also activate natural immunity. The TLR2-PI3K/AKT signaling pathway is also known to regulate allergic airway inflammation and autophagy in mouse macrophages. In wild-type mice, the expression of inflammatory factors decreased following PI3K inhibition, whereas in TLR2-KO mice, the inhibition of PI3K did not show a significant change in terms of the expression of inflammatory factors. This implies that TLR2 acts upstream of PI3K and regulates the inflammatory response (Jiang et al., 2017). Our results also confirm that PI3K was activated by TLR2 and that the inhibition of TLR2 reduces the levels of PI3K expression; however, the inhibition of PI3K had no effect on TLR2 expression. TLR2 is also able to maintain intrinsic immune barrier homeostasis via PI3K/AKT regulation, balancing intramucosal homeostasis with inflammatory stress-induced damage *in vivo* (Cario et al., 2007). Mycobacterium tuberculosis has been shown to inhibit proinflammatory cytokines and downregulate antigen-presenting cell function via the TLR2-PI3K/AKT pathway in mouse macrophages, thus weakening T-cell-mediated immune responses (Liu et al., 2016). The TLR2-PI3K/AKT signaling pathway not only exerts functionality in inflammation; it also induces cytoskeletal rearrangements and polarization in macrophage (Lasunskaia et al., 2006) and plays a crucial role in priming antigen-specific naive T cells (Cho et al., 2018). It has been suggested that CD14 assists in TLR activation under certain circumstances (Pauligk et al., 2004). CD14 was also found to be activated in our current results, although the role of CD14 in the TLR2-PI3K-AKT pathway could not be determined; this needs further investigation.

PI3K has been shown to regulate the activation of multiple intracellular signaling processes and the function of many inflammatory factors (Yang et al., 2015; Xuan et al., 2017). The PI3K/AKT signaling pathway plays a crucial role in viral infection, replication, translation, cellular processes, cancer, protein synthesis, glucose metabolism, and inflammation (Jing et al., 2016). PI3K/AKT is known to be activated by extracellular stimuli including cytokines, growth factors, G protein coupled receptors, and B cell receptors (Liu and Cohen, 2015). Phosphatase and tensin homolog (PTEN; Stambolic et al., 1998), inositol polyphosphate-4-phosphatase, type II (INPP4B), leucine-rich repeat protein phosphatase 2 (PHLPP2; Brognard et al., 2007), and protein phosphatase 2 (PP2A; Kuo et al., 2008) also known to be regulated by PI3K activity. There is also evidence to suggest that multiple viruses can activate the PI3K/AKT signaling pathway and exert differential effects. Semliki Forest virus (SFV), Ross River virus (RRV), and chikungunya virus (CHIKV) are all known to activate the PI3K/AKT signaling pathway. CHIKV activates PI3K/AKT with a lower activation efficiency than SFV and RRV while virus replication and translation are controlled by the PI3K/AKT pathway (Joubert et al., 2015; Broeckel et al., 2019). Herpesviruses (HSV) enter cells in a manner that depends on activation of the PI3K/AKT pathway; the inhibition of PI3K blocks HSV entry (Tiwari and Shukla, 2010). Considering our results, PI3K can be activated by different upstream proteins; the TLR2-activated PI3K/AKT pathway seems to be more inclined toward inflammatory responses and autophagy, whereas the viral glycoprotein-activated PI3K/AKT pathway seems to be more advantageous for viral infection and long-term presence. These hypotheses need to be tested in the future. Another thing that deserves to be noticed is that PI3K is the central regulatory molecule that activates AKT. However, it has been shown that other kinases can also activate AKT directly. Ack1 (one of the Non-receptor tyrosine kinases) can directly regulate Akt by forming isoforms with AKT (Mahajan and Mahajan, 2010). Protein tyrosine kinase 6 (PTK6) and Src mediated AKT phosphorylation directly, rather than PI3K (Chen et al., 2001; Zheng et al., 2010). Overall, multiple tyrosine kinases activate AKT in a PI3K-independent manner by phosphorylating AKT at multiple tyrosine residues (Mahajan and Mahajan, 2012). These results suggest that PI3K activation is only one of the AKT signaling pathways. More research is needed to explore the PI3K-AKT gaps. These may play a broad suggestive role in future studies of the PI3K-AKT signaling pathway.

In our present study, we found that TLR2 expression was reduced at 24 hpi. The possible reason for this is that the reduction in TLR2 surface expression after infection is due to the release of soluble proteins; this results in higher levels of soluble TLR2 under certain infectious and inflammatory processes (Henrick et al., 2016; Holst et al., 2017; Hossain et al., 2018). On the other hand, the expression of viral proteins at different locations in the cell has different effects on TLR2 activation. On the premise that the S protein of SARS-CoV-2 is known to activate TLR2, the expression of the S protein in the cytoplasm and on the cell surface activates TLR2 in macrophages and causes natural immune activation, while the expression of TLR2 outside the cell does not activate TLR2. This supports our speculation of diminished TLR2 activation 24 h after JEV infection (Khan et al., 2021).

Japanese encephalitis virus infection has been demonstrated to activate TLR3 and RIG-I, thus resulting in the regulation of the NF-κB pathway; this activates microglia involvement in inflammatory responses in the CNS (Jiang et al., 2014). TLRs trigger several distinct signaling pathways to produce pro-inflammatory cytokines and interferon genes (Bagchi et al., 2007; Kenny et al., 2009). According to our results, TLR2 functions through the PI3K-AKT signaling axis. One possibility is that the JEV strain we used has its own TLR2 activation mechanism. A previous study revealed that TLR2 involvement is independent of DENV’s replicative potential and is dependent on viral particle recognition (Aguilar-Briseño et al., 2020). As a result, TLR2 activation might be caused by alterations of proteins in the JEV strain. However, no conclusive evidence exists to demonstrate that TLR2 activation is not dependent on JEV replication and is instead dependent on structural proteins, although this requires additional investigation.

In summary, using a proteomic approach, we investigated the *in vitro* time-course of JEV infection in BV2 cells and discovered for the first time that JEV activates inflammatory responses via the TLR2-PI3K-AKT axis. Our study provides further understanding of the molecular pathways underlying JEV-induced inflammatory cytokines. Our findings ultimately lay the foundation for the control of JEV-induced inflammatory responses and the avoidance of intense inflammation-induced brain damage.

## Data availability statement

The data presented in the study are deposited in the EBI-PRIDE repository, accession numbers PXD035938 and PXD033575.

## Author contributions

GZ, HL, and NJ conceived the study. GZ, JZ, and YG contributed to data collection, analysis, and interpretation. HZ, FN, and CX helped with data visualization. GZ and YZ completed the drafting of the manuscript. ZH, XZ, PZ, and ZL revised the manuscript. CL and SF supervised the research. All authors contributed to the article and approved the submitted version.
