# Supplementary material for: Toll-like receptor 2 signaling pathway activation contributes to a highly efficient inflammatory response in Japanese encephalitis virus-infected mouse microglial cells by proteomics
Source: Front Microbiol. 2022 Sep 12;13:989183. doi: 10.3389/fmicb.2022.989183 (PMC9511957; doi:10.3389/fmicb.2022.989183)
Supplement: Supplementary file 3 [file Data_Sheet_1.doc]

SUPPLEMENTARY MATERIAL 1

Content 1:

**Material and methods**

**Protein Extraction and Trypsin Digestion**

1% protease inhibitor (Merck Millipore) was added into each group of cells, the samples were ultrasonic lysis 5s on and then 5s off for 10 min in ultrasonic lysis machine. Centrifuge at 12000 g for 10 min. The supernatant was transferred to a new centrifugal tube, all these steps with 4 ℃ environment. Protein concentration was determined using BCA kit (Beyotime Biotechnology) and samples keep in -80℃ for follow-on operational test.

After protease hydrolysis and then the addition of pre-cooled acetone, precipitation, centrifugation, and sonication, trypsin (Promega) was added at a ratio of 1:50 (protease: protein, m/m). Add dithiothreitol (DTT, Sigma-Aldrich) to a final concentration of 5 mM, and reduce it to 56 ℃ for 30 min. Then add iodoacetamide (IAA, Sigma-Aldrich) to make the final concentration 11 mM, and incubate for 15 min at room temperature in the dark.

**HPLC classification**

The peptides were fractionated by high-pH reversed-phase HPLC (Agilent), and the column was Agilent 300Extend C18 (5 μm particle size, 4.6 mm inner diameter, 250 mm length). Step gradient of peptide was 8%-32% acetonitrile, pH=9, separated 60 components in 60 minutes, then the peptides were combined into 42 components. The combined components were vacuum freeze-dried for subsequent operations.

**TMT mark**

The peptides digested by trypsin were desalted with Strata X C18 (Phenomenex) and then freeze-dried in vacuo. Dissolve the peptides with 0.5 M TEAB (Sigma-Aldrich), and label the peptides according to the instructions of the TMT kit (Thermo scientific). The simple operation is as follows: the labeling reagent is thawed and dissolved in acetonitrile, mixed with the peptide and incubated for 2 h at room temperature, the labeled peptide is mixed and desalted, and vacuum freeze-dried.

**LC-MS/MS Analysis**

Liquid chromatography-mass spectrometry analysis

The peptides are dissolved in the mobile phase A of liquid chromatography and then separated using the EASY-nLC 1200 ultra-high performance liquid system (Thermo scientific). Mobile phase A is an aqueous solution containing 0.1% formic acid and 2% acetonitrile; mobile phase B is an aqueous solution containing 0.1% formic acid and 90% acetonitrile. Liquid gradient setting: 0-38 min, 6%~22%B; 38-52 min, 22%~32%B; 52-56 min, 32%~80%B; 56-60 min, 80%B, and flow rate is maintained at 500 nL/min. The peptides are separated by the ultra-high performance liquid system and then injected into the NSI ion source for ionization and then analyzed by Q ExactiveTM HF-X mass spectrometry (Thermo scientific). The ion source voltage is set to 2.1 kV, and the peptide precursor ions and their secondary fragments are detected and analyzed by high-resolution Orbitrap. The scanning range of the primary mass spectrum is set to 350-1600 m/z, and the scanning resolution is set to 120,000; the scanning range of the secondary mass spectrum is set to a fixed starting point of 100 m/z, and the secondary scanning resolution is set to 30000. The data acquisition mode uses the data-dependent scanning (DDA) program, that is, after the first-level scan, the first 20 peptide precursor ions with the highest signal intensity are selected to enter the HCD collision cell and use 28% of the fragmentation energy for fragmentation. Two-stage mass spectrometry analysis is also carried out sequentially. In order to improve the effective utilization of the mass spectrometer, the automatic gain control (AGC) is set to 3E6, the signal threshold is set to 8.3E4 ions/s, the maximum injection time is set to 50ms, and the dynamic rejection time of the tandem mass spectrometry scan is set to 30s to avoid repeated scanning of ions.

**Database Search and Bioinformatics Analysis**

The resulting MS/MS data were processed using MaxQuant search engine (v.1.6.15.0). Tandem mass spectra were searched against the Mus_musculus database (17063 entries) concatenated with reverse decoy database. Trypsin/P was specified as cleavage enzyme allowing up to 2 missing cleavages. The mass tolerance for precursor ions was set as 20 ppm in First search and 5 ppm in Main search, and the mass tolerance for fragment ions was set as 0.02 Da. Carbamidomethyl on Cys was specified as fixed modification. Acetylation on protein N-terminal, oxidation on Met and deamidation (NQ) were specified as variable modifications. TMT-6plex quantification was performed. FDR was adjusted to < 1% and minimum score for peptides was set > 40

InterProScan software was used to annotate the function of the proteins by Gene Ontology (GO, http://www.ebi.ac.uk/interpro/) according to the protein sequence alignment method. Then, the proteins were classified based on the three GO annotation categories: biological process, cellular component and molecular function. Furthermore, functional enrichment analysis was performed on the basis of the GO and Kyoto Encyclopedia of Genes and Genomes (KEGG, http://www.genome.jp/kaas-bin/kaas_main) pathway analyses, and the corresponding functions and pathways with p < 0.05 (two-tailed Fisher’s exact test) were considered statistically significant.

Quantification of Differentially Expressed Proteins (DEPs) by PRM

For the JEV-infected group, 15 upregulated DEPs were selected for validation by PRM. Protein extraction and tryptic digestion were performed following the same protocol used for the label-free quantitative proteomics experiment.

PRM mass spectrometric analysis was performed on a Q ExactiveTM Plus mass spectrometer (Thermo, USA) coupled online to a UPLC (Thermo scientific). The peptides are dissolved in the mobile phase A of liquid chromatography and then separated using the EASY-nLC 1200 ultra-high performance liquid system. Mobile phase A is an aqueous solution containing 0.1% formic acid and 2% acetonitrile; mobile phase B is an aqueous solution containing 0.1% formic acid and 90% acetonitrile. Liquid gradient setting: 0-16 min, 7%~25%B; 16-22 min, 25%~35%B; 22-26 min, 35%~80%B; 26-30 min, 80%B, and flow rate is maintained at 500 nL/min. The electrospray voltage was applied at 2.1 kV. The m/z scan ranged from 300 to 1040 for a full scan, and intact peptides were detected in the Orbitrap at a resolution of 70000. Peptides were then selected for MS/MS using an NCE setting of 27, and the fragments were detected in the Orbitrap at a resolution of 17500. AGC was set at 3E6 for full MS and 1E5 for MS/MS. The maximum IT was set at 50 ms for full MS and 200 ms for MS/MS. The isolation window for MS/MS was set at 1.6 m/z. The resulting MS data were processed using Skyline (v.3.6). After normalizing the quantitative information, relative quantitative analysis was performed on the target peptides.

Content 2:

The qPCR primers used in this study

| Target gene |  | Primers (5′ -3′) |
| --- | --- | --- |
| GAPDH | Forward | CTCAACTACATGGTCTACATGTTC |
|  | Reverse | ATTTGATGTTAGTGGGGTCTCGCTC |
| TRIF | Forward | AACCTCCACATCCCCTGTTTT |
|  | Reverse | GCCCTGGCATGGATAACCA |
| TIRAP | Forward | CCTCCACTCCGTCCAAGAAG |
|  | Reverse | TGAACCATCATAGAGGTGGCTTT |
| TRAM | Forward | CGATCAAGACGGCCATGAGTC |
|  | Reverse | CTCGTCGGTGTCATCTTCTGC |
| TLR1 | Forward | TGAGGGTCCTGATAATGTCCTAC |
|  | Reverse | AGAGGTCCAAATGCTTGAGGC |
| TLR2 | Forward | GCAAACGCTGTTCTGCTCAG |
|  | Reverse | AGGCGTCTCCCTCTATTGTATT |
| TLR3 | Forward | GTGAGATACAACGTAGCTGACTG |
|  | Reverse | TCCTGCATCCAAGATAGCAAGT |
| TLR4 | Forward | ATGGCATGGCTTACACCACC |
|  | Reverse | GAGGCCAATTTTGTCTCCACA |
| TLR5 | Forward | GCAGGATCATGGCATGTCAAC |
|  | Reverse | ATCTGGGTGAGGTTACAGCCT |
| TLR6 | Forward | TGAGCCAAGACAGAAAACCCA |
|  | Reverse | GGGACATGAGTAAGGTTCCTGTT |
| TLR7 | Forward | ATGTGGACACGGAAGAGACAA |
|  | Reverse | GGTAAGGGTAAGATTGGTGGTG |
| TLR8 | Forward | GAAAACATGCCCCCTCAGTCA |
|  | Reverse | CGTCACAAGGATAGCTTCTGGAA |
| TLR9 | Forward | ATGGTTCTCCGTCGAAGGACT |
|  | Reverse | GAGGCTTCAGCTCACAGGG |
| TNF-α | Forward | CAGGCGGTGCCTATGTCTC |
|  | Reverse | CGATCACCCCGAAGTTCAGTAG |
| CCL3 | Forward | TGTACCATGACACTCTGCAAC |
|  | Reverse | CAACGATGAATTGGCGTGGAA |
| iNOS | Forward | GTTCTCAGCCCAACAATACAAGA |
|  | Reverse | GTGGACGGGTCGATGTCAC |
